# Regulation of the abundance of Y-family polymerases in the cell cycle of budding yeast in response to DNA damage

**DOI:** 10.1007/s00294-020-01061-3

**Published:** 2020-02-19

**Authors:** Aleksandra Sobolewska, Agnieszka Halas, Michal Plachta, Justyna McIntyre, Ewa Sledziewska-Gojska

**Affiliations:** Institute of Biochemistry and Biophysics, Polish Academy of Sciences, ul. Pawinskiego 5A, 02-106 Warsaw, Poland

**Keywords:** Polymerase eta, Rev1, Y-family polymerases, S-phase checkpoint, DNA damage response, *S. cerevisiae*

## Abstract

Y-family DNA polymerases mediate DNA damage tolerance via translesion synthesis (TLS). Because of the intrinsically error-prone nature of these enzymes, their activities are regulated at several levels. Here, we demonstrate the common regulation of the cellular abundance of Y-family polymerases, polymerase eta (Pol eta), and Rev1, in response to DNA damage at various stages of the cell cycle. UV radiation influenced polymerase abundance more when cells were exposed in S-phase than in G1- or G2-phases. We noticed two opposing effects of UV radiation in S-phase. On one hand, exposure to increasing doses of UV radiation at the beginning of this phase increasingly delayed S-phase progression. As a result, the accumulation of Pol eta and Rev1, which in nonirradiated yeast is initiated at the S/G2-phase boundary, was gradually shifted into the prolonged S-phase. On the other hand, the extent of polymerase accumulation was inversely proportional to the dose of irradiation, such that the accumulation was significantly lower after exposure to 80 J/m^2^ in S-phase than after exposure to 50 J/m^2^ or 10 J/m^2^. The limitation of polymerase accumulation in S-phase-arrested cells in response to high UV dose was suppressed upon *RAD9* (but not *MRC1*) deletion. Additionally, hydroxyurea, which activates mainly the Mrc1-dependent checkpoint, did not limit Pol eta or Rev1 accumulation in S-phase-arrested cells. The results show that the accumulation of Y-family TLS polymerases is limited in S-phase-arrested cells due to high levels of DNA damage and suggest a role of the Rad9 checkpoint protein in this process.

**Electronic supplementary material:**

The online version of this article (10.1007/s00294-020-01061-3) contains supplementary material, which is available to authorized users.

## Introduction

Translesion DNA synthesis (TLS) plays a significant role in rescuing DNA replication which very often, at least once in each DNA replication round, arrests at sites of DNA damage caused by genotoxic agents or spontaneous DNA decay (Fuchs and Baynton 2000). This DNA damage tolerance pathway relies on specialized TLS polymerases whose active centers can tolerate noncanonical DNA base pairs during replication (Prakash et al. 2005). The ability to accommodate damaged DNA bases and a lack of exonucleolytic proofreading activity typical of regular DNA-replicating enzymes make TLS polymerases intrinsically error prone. Consequently, appropriate regulation of the cellular abundance, activities, and targeting of TLS polymerases is important for the maintenance of genome integrity.

The majority of TLS polymerases belong to the Y-family of DNA polymerases. Two members of this family, Rev1 and polymerase eta (Pol eta), function in budding yeast. Pol eta is one of the founding members of the Y-family polymerases (Ohmori et al. 2001) and is exceptional among eukaryotic TLS polymerases in its ability to efficiently and accurately bypass cyclobutane pyrimidine dimers (CPDs), the major type of DNA lesions induced by UV light (Johnson et al. 1999a, b, c; Masutani et al. 1999). Consequently, defects in the activity of this polymerase lead to increased UV-induced mutagenesis in yeast and higher eukaryotes and result in a variant form of xeroderma pigmentosum syndrome that predisposes humans to skin cancer (Johnson et al. 1999a, b, c). In addition to CPD, Pol eta is involved in bypassing major mutagenic oxidative DNA damage, 7,8-dihydro-8-oxoguanine (Carlson and Washington 2005), and other replication-blocking lesions. For some of these lesions, Pol eta-mediated TLS is less efficient than for others and may be highly inaccurate (Bresson 2002). Pol eta is also extremely error prone when replicating undamaged DNA template (Washington et al. 1999; McCulloch and Kunkel 2008). Additionally, there is growing evidence based on experiments with different organisms, suggesting that Pol eta and other Y-family polymerases also exhibit TLS-independent cellular functions (Acharya et al. 2019; Henrikus et al. 2018; McIntyre 2020).

The second yeast Y-family polymerase, Rev1, is a deoxycytidyl transferase that uses an internal arginine as a template to preferentially incorporate cytosine (Nair 2005) across regular or damaged DNA bases and abasic sites (Garg et al. 2005; Nelson et al. 2000). In addition to this unique enzymatic activity, Rev1 plays a more general function in TLS as a scaffold protein for other TLS polymerases. In both yeast and mammalian cells, Rev1 functionally and physically interacts with B-family TLS polymerase zeta (Pol zeta) (Acharya et al. 2006), which is important for the majority of Pol zeta-mediated spontaneous and DNA damage-induced mutagenesis in dividing (Prakash et al. 2005; Haracska et al. 2001a, b; Niu et al. 2019; Szwajczak et al. 2018) as well as stationary yeast cells (Halas et al. 2009). Additionally, Rev1 interacts with other Y-family polymerase members in both yeast (Acharya et al. 2007) and higher eukaryotes (Guo et al. 2003; Ohashi et al. 2004; Kosarek et al. 2008), and these interactions facilitate the recruitment of these polymerases to the DNA replication machinery and stimulate TLS mechanisms engaging more than one polymerase.

Because of the potentially mutagenic character of TLS polymerases, their access to the replication machinery should be restricted to DNA synthesis-perturbing events. The main scaffold protein that orchestrates members of the replication complex is PCNA, which forms a trimeric DNA replication processivity clamp. Pol eta and Rev1 interact with PCNA in two distinct ways, and both of these interactions stimulate the enzymatic activity of the polymerases (Haracska et al. 2001a, b; Sharma et al. 2011). In response to replication stress, PCNA is ubiquitinated at Lys^164^ by the Rad6/Rad18 complex (Hoege et al. 2002), and this modification specifically facilitates the interaction of Y-family polymerases with PCNA and positions them in the replication fork (Garg and Burgers 2005). Pol eta and Rev1 interact with ubiquitinated PCNA via their C-terminal ubiquitin binding motifs, UBZ and UBM, respectively, and the presence of these motifs has been shown to stimulate TLS (Bienko et al. 2005). Rev1 has also been shown to interact with a member of the SWI/SNF superfamily, Rad5. This interaction is important for the noncatalytic function of Rev1 in TLS and crucial for Rev1-dependent repair during both physiological and induced base-damage-free replication stress leading to the accumulation of single-stranded DNA gaps (Gangavarapu et al. 2006; Kuanga et al. 2013; Xu et al. 2016; Fan et al. 2018; Gallo et al. 2019). In addition, cellular responses to DNA damage and replication stress in eukaryotes are coordinated by the activation of cell cycle checkpoints (Weinert 1998). The role of checkpoint activation in the regulation of TLS is far from being fully understood. However, in yeast defective in nucleotide excision repair (NER), UV-induced mutagenesis relying on Pol zeta and Rev1 activities has been reported to be affected by *MEC1* deletion (Pagès et al. 2009). Mec1 kinase functions as a sensor of DNA damage and transmits the checkpoint signal by phosphorylating the downstream effector kinase Rad53 (Nyberg et al. 2002; Paulsen and Cimprich 2007). Additionally, Mec1-dependent phosphorylation promotes the association of the Rev1-Pol zeta complex with regions near DNA double-strand breaks in a PCNA ubiquitination independent manner (Hirano and Sugimoto 2006).

Regulation of the cellular abundance of TLS polymerases appears to be another significant, although much less explored, factor influencing their activities. A global analysis of the status of Pol eta in skin cancer cells demonstrated that, in a cohort of patients showing no changes in the sequence of the Pol eta coding gene, the *POLH* mRNA expression was either decreased or increased in skin tumor tissue compared with normal tissue (Flanagan et al. 2007). It has also been shown that abnormal upregulation of human Pol eta through interferon regulatory factor 1 (IRF1) transactivation is responsible for the increase in mutation frequency and carcinogenesis in cells exposed to the alkylating agent *N*-methyl-*N′*-nitro-*N*-nitrosoguanidine (Qi et al. 2012). In turn, upregulation of *hREV1* has been shown to be associated with the pathogenesis of human glioma (Wang et al. 2010). Overexpression of this polymerase also promotes the accumulation of point mutations and the development of carcinogen-induced intestinal adenomas (Sasatani et al. 2017). In *Saccharomyces cerevisiae*, overproduction of Pol eta increases the frequency of spontaneous mutations, especially in mutant strains deficient in either the 3′ → 5′ proofreading exonuclease activity of polymerase epsilon or mismatch repair (Pavlov et al. 2001). Together, these results indicate that the misregulation of Y-family polymerases can compromise genome integrity.

Most information on the regulation of the cellular abundance of Y-family DNA polymerases comes from studies on yeast (Skoneczna et al. 2007; Waters and Walker 2006; Plachta et al. 2015). The cellular levels of both Pol eta and Rev1 have been shown to be cell cycle-regulated under normal, stress-free conditions. The levels of these polymerases are lowest at the G1 and G1/S stages of the cell cycle, peaking in G2-phase (Plachta et al. 2015; Waters and Walker 2006). It has been postulated that this pattern of regulation is associated with the role of Y-family polymerases in TLS, which occurs predominantly in G2-phase in yeast (D’Souza et al. 2008; Waters and Walker 2006). However, limited data about the regulation of the abundance of these polymerases in response to agents that cause DNA damage at various cell cycle stages are available.

Here, we analyzed the regulation of Pol eta and Rev1 abundance in response to either UV radiation at various stages of the cell cycle, which produces DNA lesions (bypass of which can involve Pol eta and Rev1 in different ways), or hydroxyurea (HU) treatment, which causes replication stress by limiting dNTP pools. Our results show the common regulation of Y-family DNA polymerase abundance in the cell cycle in response to various DNA insults. We also indicate the effect of Rad9 on polymerase accumulation in cells arrested is S-phase in response to high doses of UV radiation.

## Materials and methods

### Yeast strains

Analyses of mRNA, protein levels and survival were performed in *S. cerevisiae* haploid strains derived from the BY4741 (*MATa his3Δ leu2Δ met15Δ ura3*) strain (Open Biosystem). Mutagenesis assays were performed in derivatives of CL1265-7C (Morrison et al. 1989). Strains BY*Rev1-ProA-His*_*7*_, *mrc1*Δ *Rev1-ProA-His7*, *rad9*Δ *Rev1-ProA-His*_*7*_, and CL1265-7C *Rev1-ProA-His*_*7*_ carrying Rev1 C-terminally tagged with a TEV–ProA–His_7_ epitope (marked with *HIS3*) were constructed by direct transformation of BY4741, *mrc1*Δ and *rad9*Δ or CL1265-7C cells, respectively, using a PCR-amplified cassette derived from DNA extracted from the YLW70 strain (*REV1-ProA- His*_*7*_), which was kindly provided by G.C. Walker (Wiltrout and Walker 2011b). Co-immunoprecipitation assay was performed with extracts from strains DGY19 (*MATa RAD5-6His10FLAG::kanMX6 leu2Δ0 his3Δ1 ura3Δ0 met15Δ0*), which was kindly provided by G. W. Brown (Gallo et al 2019) and derivative of BY4741 carrying *Rev1-ProA-His7*.

### Cell synchronization

Yeast strains were grown in YPD medium at 23 °C until they reached an OD_600_ of 0.2–0.3. Cell growth was arrested in G1-phase by treatment with α factor for 2.5 h at the same temperature. The α factor was added to the culture in two doses at 2 µg/ml, first at the beginning of synchronization and then after 75 min of incubation. For cell cycle progression experiments, the α factor was washed away three times with water, and the cells were resuspended in fresh YPD medium. For G2/M arrest, the yeast cultures were synchronized for 2.5 h with 20 µg/ml nocodazole.

### UV and HU treatment

Yeast cells synchronized with α factor or nocodazole were harvested by centrifugation, washed twice with water, and centrifuged again. For UV treatment, the pellets were resuspended in 10 ml of water, and the cells were irradiated on glass Petri dishes. Then, the same volume of 2 × YPD medium was added, and the cells were incubated further for the indicated times. For G1- or G2/M-phase experiments, all media and washing water were supplemented with α factor or nocodazole, respectively. For survival and mutagenesis, experiments cells irradiated in water (10 ml) were harvested by centrifugation, resuspended in water (1 ml, resulting in a 10 × concentrated suspension), and plated (in duplicate) on complete minimal medium devoid of arginine. The plates were incubated for 3–4 days at 30 °C before the mutant colonies were counted. To estimate the total number of colony-forming units, serially diluted cultures were plated on fully supplemented minimal agar plates and counted after 3 days of incubation. In each experiment, 6–10 independent cultures of the tested yeast strain were analyzed. HU was added at a final concentration of 150 mM to synchronized yeast cultures.

### Western blotting

Whole-cell extracts were prepared as previously described (Skoneczna et al. 2007). A rabbit peroxidase-anti-peroxidase affinity-isolated antibody (PAP, Sigma-Aldrich) was used to detect the protein A tag; a monoclonal anti-Pgk1 primary antibody (Invitrogen) and an anti-mouse HRP-conjugated immunoglobulin (IgG) secondary antibody (Dako) were used to detect Pgk1; a goat anti-Rad30 primary antibody (Santa Cruz) and donkey anti-goat HRP-conjugated IgG secondary antibody (Santa Cruz) were used to detect Pol eta. Blotting to detect native Pol eta was performed as previously described (Plachta et al. 2015). Briefly, the membrane was blocked overnight at 4 °C in a mixture of equal portions (1:1) of non-animal protein (NAP) blocker (G Biosciences) and Tris-buffered saline with Tween 20 (TBST). Then, the blot was incubated for 2 h at room temperature in a mixture of equal volumes of SC-11868 and SC-11866 anti-Rad30 antibodies. This incubation step was followed by incubation in a solution of secondary HRP IgG for 2 h. The signal was detected using chemiluminescent substrates for HRP (SuperSignal West Pico, Thermo Scientific) or, in the case of native Pol eta, West Femto substrate (Thermo Scientific) using a charge-coupled device (CCD) gel imager. The resulting bands were quantified using ImageJ 1.47 software (NIH, USA). The quantification procedure always included normalization to the levels of Pgk1. The loading control and target protein band intensities were quantified within the linear range of detection (unsaturated western blot signal).

### Co-immunoprecipitation

Whole yeast cell extracts were obtained from 100 ml of cultures of DGY19 strain and BY*Rev1-ProA-His*_*7*_. Cells of the latter one were synchronised with nocodazole. Cells were washed with water and resuspended in 1 ml of RIPA lysis buffer [25 mM Tris–HCl, pH 7.6, 150 mM NaCl, 1% Non-ident P-40, 1 mM EDTA, 1 mM PMSF, 1 mM Na_3_VO_4_, and protease inhibitor cocktail (Sigma)], followed by disruption with beating beads for 15 min and centrifugation for 20 min at 4 °C. Total cellular yeast extracts containing 2 mg of protein were incubated with 40 µl of 50% anti-FLAG affinity gel (Bimake.com) with rotation at 4 °C overnight. The resins were washed three times with 1 ml of lysis buffer and, together with appropriate controls, analyzed by SDS-PAGE and western blot. To detect FLAG-Rad5, mouse anti-FLAG primary (Origene) and goat anti-mouse Alexa546 (Invitrogen) secondary antibodies were used. Rabbit peroxidase–anti-peroxidase affinity–isolated antibody (PAP, Sigma-Aldrich) were used to detect Rev1-ProA protein.

### Total RNA extraction and real-time quantitative PCR (RT-qPCR) analysis

Exponentially growing yeast cells (1–2 × 10^7^ cells/ml) were harvested by centrifugation, washed with water, and centrifuged again. The supernatant was immediately aspirated, and the cells were frozen in liquid nitrogen and stored at − 80 °C. Total RNA was extracted by the hot acid phenol method, as described at https://younglab.wi.mit.edu/expression/totalRNAprep.html. Removal of contaminating genomic DNA and reverse transcription (RT) using 1 μg of total RNA as a template were performed using a Maxima First-Strand cDNA Synthesis RT-qPCR kit with DNase (Thermo Scientific) according to the manufacturer’s recommendations. Control PCR was performed without prior RT to ensure that the RNA samples were not contaminated with genomic DNA. The reactions were performed with RT HS-PCR mix SYBR^®^ A (A&A Biotechnology, Poland) and a LightCycler^®^ 480 II system (Roche) according to the manufacturer’s protocol. The following primers were used for RT-qPCR (for *ACT1* [to normalize the data], *RAD30* and *REV1*): *ACT1* forward ACCGCTGCTCAATCTTCTTC and *ACT1* reverse GTAGTTTGGTCAATACCGGC; *RAD30* forward GCCTTTTTTGCACAGGTTGAG and *RAD30* reverse CGCAGACTACCGGATCTTCTT; *REV1* forward GCGAAAAGGATAGTCGCTTG and *REV1* reverse CTTCCATGCGGAGAGATGAT). The amplification curves were analyzed using Roche LC software (version 1.5) to both determine crossing point Cp values (by the second derivative method) and perform melting curve analysis. RT-qPCR was performed with at least five biological and three technical replicates.

## Results

### UV irradiation affects the cellular abundance of Y-family polymerases in S-phase

Previous findings that the TLS polymerases Pol eta and Rev1 are regulated in the cell cycle under stress-free conditions (Waters and Walker 2006; Plachta et al. 2015) prompted us to further investigate the regulation of these polymerases in response to DNA damage. Since it has previously been shown that tagging of Pol eta with various epitopes differently affects the stability of this polymerase (Plachta et al. 2015), we analyzed the levels of the native form of Pol eta in the current study. On the other hand, we studied the levels of a Rev1-ProA fusion protein, as an assay for immunodetection of the native form of Rev1 in yeast extracts has not yet been developed. Although Rev1-ProA was previously characterized and is considered reliable, recent evidence has shown that the C-terminus of Rev1 interacts with Rad5, which is essential for the DNA damage response (Gallo et al. 2019; Xu et al. 2016). Therefore, to determine whether attachment of the ProA epitope to the C-terminus of Rev1 affects the interaction between Rev1 and Rad5, we performed co-immunoprecipitation assay between Rad5 protein tagged with FLAG epitope and Rev1-ProA protein. Additionally, we compared the roles of Rev1 and Rev1-ProA in UV-induced reversion of the *arg4-17* mutation, which has previously been shown to depend on the interaction between Rev1 and Rad5 (Kuang et al. 2013). Since our results showed that Rad5-FLAG and Rev1-ProA proteins interact and no significant difference in reversion frequency between cells producing native Rev1 and cells producing Rev1-ProA (Supplementary material Fig. S1a and b), we decided to use the tagged form of Rev1 in further experiments.

**Fig. 1:**
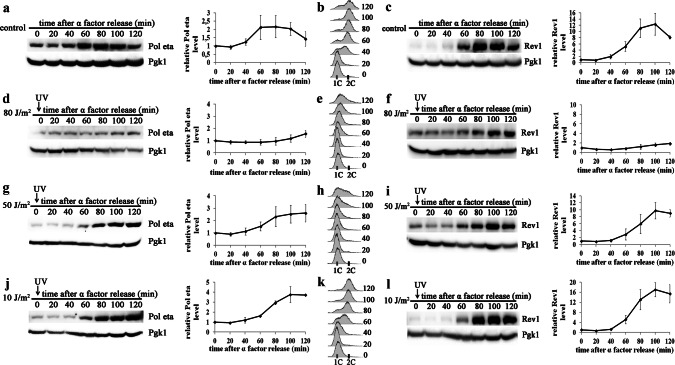
Levels of Pol eta and Rev1 in yeast irradiated with UV light at various doses at entry into S-phase. Extracts from cells released from α factor arrest and harvested at the indicated time points were probed with antibody against Pol eta (**a**, **d**, **g**, **j**) or Rev1-ProA (**c**, **f**, **i**, **l**) and antibody against Pgk1 as a loading control. Cells entering S-phase grew untreated (control; **a**, **b**, **c**) or were immediately irradiated with 80 J/m^2^ (**d**, **e**, **f**), 50 J/m^2^ (**g**, **h**, **i**), and 10 J/m^2^ (**j**, **k**, **l**). **a**, **c**, **d**, **f**, **g**, **i**, **j**, **l** Western blots from representative experiments (left); the results are the mean quantitative values from western blots from three to four independent experiments ± SDs (right). The band intensities were normalized to the intensities of the respective Pgk1 bands and to intensities at time 0′. **b**, **e**, **h**, **k** FACS data were used to monitor S-phase progression

To analyze the effect of UV radiation in S-phase on the abundance of Pol eta and Rev1, yeast cells arrested in G1-phase were released into S-phase progression by the removal of the α factor and irradiated with 80 J/m^2^ at the beginning of S-phase (time 0′). Consistent with previously published results (Plachta et al. 2015; Waters and Walker 2006), a two-to-threefold increase in Pol eta and a 10-to-12-fold increase in Rev1 accumulation were observed in control (untreated) cultures due to the progression of the cell cycle from S-phase into G2/M (Fig. 1a–c). Irradiation of the yeast cells at the beginning of S-phase with 80 J/m^2^, which had a moderate lethal effect (48.2 ± 3.5% survival, Supplementary material Fig. S2), largely inhibited cell cycle progression and the accumulation of both Pol eta and Rev1 over time (Fig. 1d–f and Supplementary Material Fig. S3). The results suggested that polymerase accumulation was inhibited in S-phase extended after exposure to UV radiation. Intriguingly, it has previously been shown that UV irradiation of yeast released from α factor arrest does not significantly affect the pattern of Rev1 polymerase accumulation (Waters and Walker 2006). Since the previous results were obtained with lower UV doses, we investigated the accumulation of Rev1 and Pol eta in cells treated with 10 and 50 J/m^2^. In yeast irradiated with 10 J/m^2^ at the onset of S-phase (time 0′), the levels of both polymerases increased over the two hours of the experiment by almost fourfold for Pol eta and 17-fold for Rev1 (Fig. 1j, l) and were slightly higher than those detected in untreated control cells in G2-phase (Fig. 1a, c). UV irradiation did not significantly affect the time course of polymerase accumulation, as this accumulation in both control and irradiated cultures began approximately 60 min after the cells entered S-phase (compare Fig. 1a and j for Pol eta and 1c and l for Rev1). However, an approximately 20-min delay in the exit from S-phase due to UV exposure slightly shifted polymerase accumulation toward S-phase (compare FACS data in Fig. 1b, k). This trend was further evident in cells irradiated with 50 J/m^2^ (Fig. 1g–i). After exposure to UV at this dose, S-phase progression was significantly delayed, and the levels of both polymerases peaked when the cells were still in S-phase at approximately 100 min after treatment. While the levels of both Pol eta and Rev1 increased after irradiation with 50 J/m^2^ by up to 2.5-fold and eightfold (Fig. 1g, i), respectively, the levels after irradiation with 50 J/m^2^ were significantly lower than those after treatment with 10 J/m^2^ (Fig. 1j, l).

**Fig. 2:**
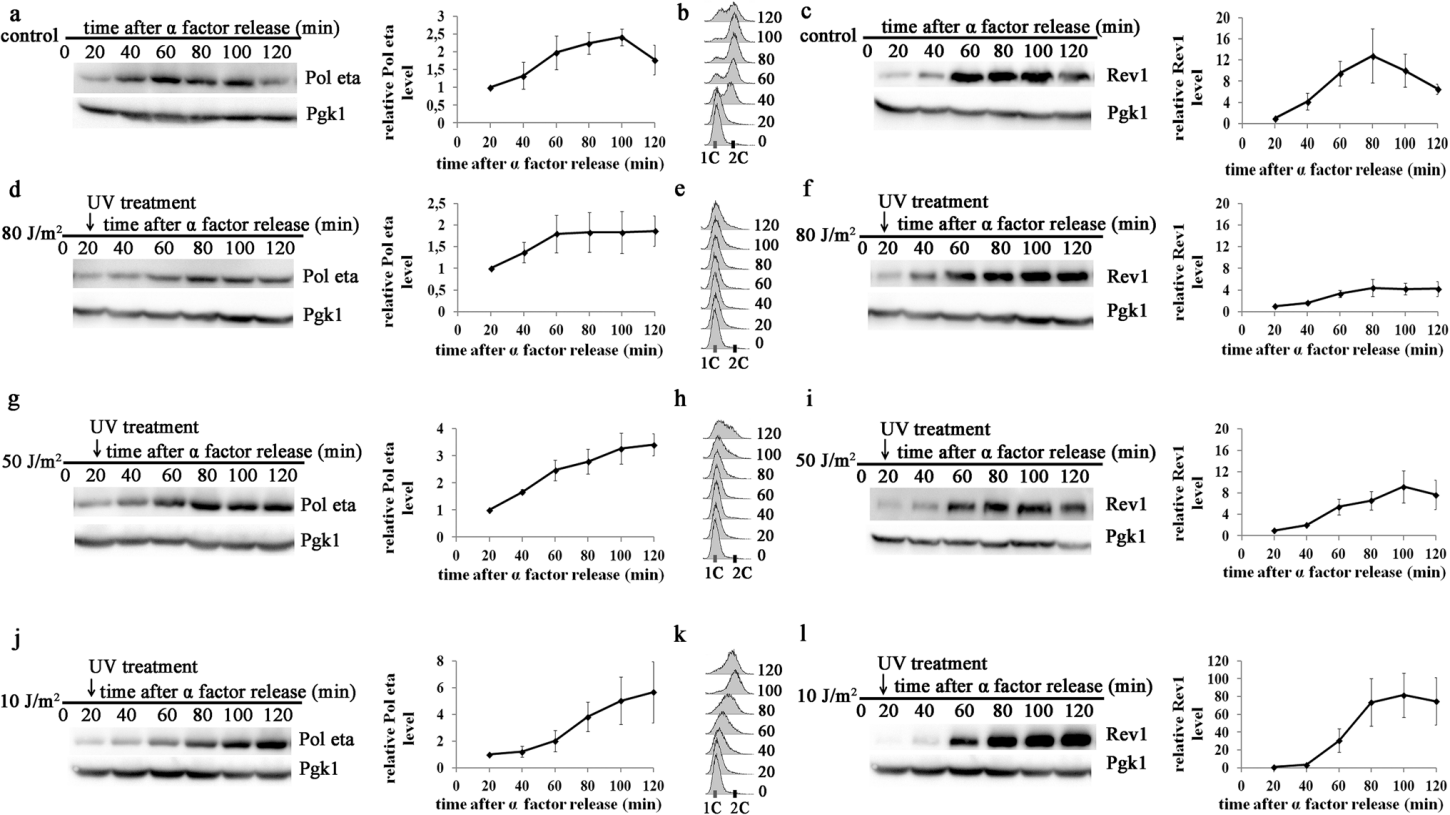
Levels of Pol eta and Rev1 in yeast irradiated with UV light at various doses in ongoing S-phase. Extracts from cells released from α factor arrest and harvested at the indicated time points were probed with an antibody against Pol eta (**a**, **d**, **g**, **j**) or Rev1-ProA (**c**, **f**, **i**, **l**) and an antibody against Pgk1 as a loading control. Cells entering S-phase grew untreated (control; **a**, **b**, **c**) or were irradiated in S-phase at 20 min after α factor removal with 80 J/m^2^ (**d**, **e**, **f**), 50 J/m^2^ (**g**, **h**, **i**), and 10 J/m^2^ (**j**, **k**, **l**). **a**, **c**, **d**, **f**, **g**, **i**, **j**, **l** Western blots from representative experiments (left); the results are the mean quantified values from western blots from three to four independent experiments ± SDs (right). Band intensities were normalized to the intensities of the respective Pgk1 bands and intensities at time 0′ after UV radiation (20′ after entry into S-phase). **b**, **e**, **h**, **k** FACS data were used to monitor S-phase progression

**Fig. 3:**
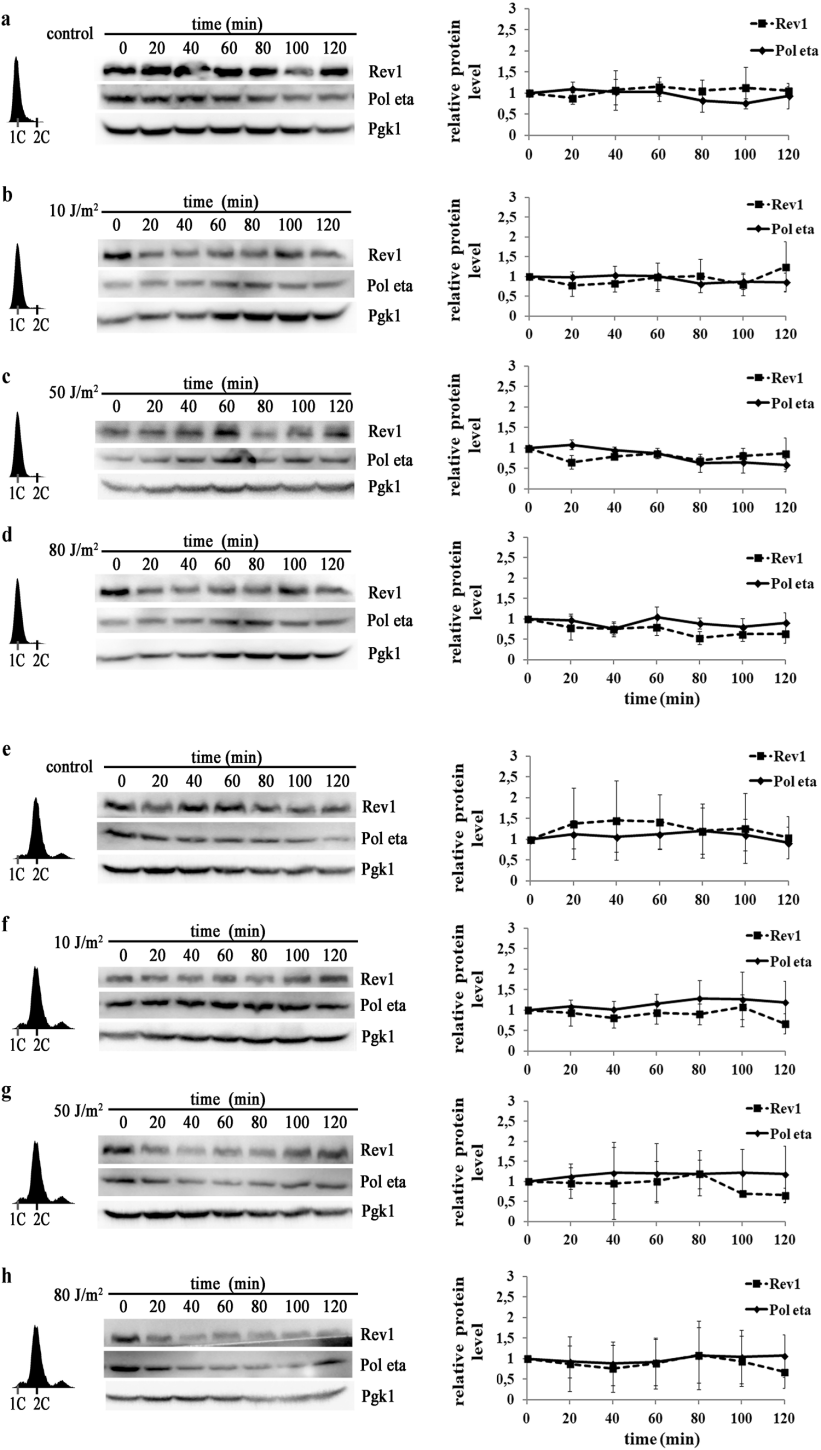
Levels of Pol eta and Rev1 in the G1- and G2/M-phases of the cell cycle after UV irradiation. Extracts from cells arrested in G1-phase with α factor (**a**, **b, c, d**) or in G2/M-phase with nocodazole (**e**, **f, g, h**) were harvested at the indicated time points after irradiation with 10 J/m^2^ (**b** and **f**), 50 J/m^2^ (**c** and **g)** or 80 J/m^2^ (**d** and **h**) (with the untreated controls shown in **a** and **e**) and probed with an antibody against Pol eta or Rev1-ProA and an antibody against Pgk1 as a loading control. FACS data indicating the cell cycle stage; western blots from representative experiments (**a**, **b**, **c**, **d, e, f, g, h** left); the results are the mean quantified values from western blots in 3–5 independent experiments ± SDs (**a**, **b**, **c**, **d, e, f, g, h** right). The band intensities were normalized to the intensities of the respective Pgk1 bands and to intensities at time 0′. Pol eta—solid line; Rev1—dashed line

Altogether, the results obtained following irradiation with various doses of UV (10, 50, or 80 J/m^2^) indicated that the levels of Y-family TLS polymerases were inversely correlated with the UV dose. Decrease in the levels of polymerase accumulation in response to increasing doses of UV radiation were also observed in yeast irradiated later in S-phase at 20 min after the removal of α factor (Fig. 2). Although the polymerase accumulation generally reached higher levels under these conditions than after irradiation at time 0′, the inverse correlation between the UV dose and polymerase levels was even more pronounced. Irradiation with 10, 50 or 80 J/m^2^ caused a five-, three- or twofold increase in the level of Pol eta, respectively (Fig. 2j, g, d). The protein levels of Rev1 increased after these treatments (10, 50 or 80 J/m^2^) by 80-, 9-, or fourfold, respectively (Fig. 3l, i, f).

It has previously been shown that under normal, stress-free conditions, the levels of both Pol eta and Rev1 are lowest in cells arrested in G1-phase of the cell cycle (Plachta et al. 2015; Waters and Walker 2006). Our results indicated that the levels of these proteins did not significantly increased when cells arrested with α factor in G1-phase were treated with UV radiation (10, 50, or 80 J/m^2^) (Fig. 3a–d). Similarly, the levels of neither Pol eta nor Rev1 were significantly affected by UV irradiation of cells arrested in G2/M due to nocodazole treatment (Fig. 3e–h), indicating that regulation of polymerase accumulation in response to UV radiation is specific to the treatment in S-phase.

### *RAD30* and *REV1* mRNA accumulate after UV radiation in S-phase.

**Fig. 4:**
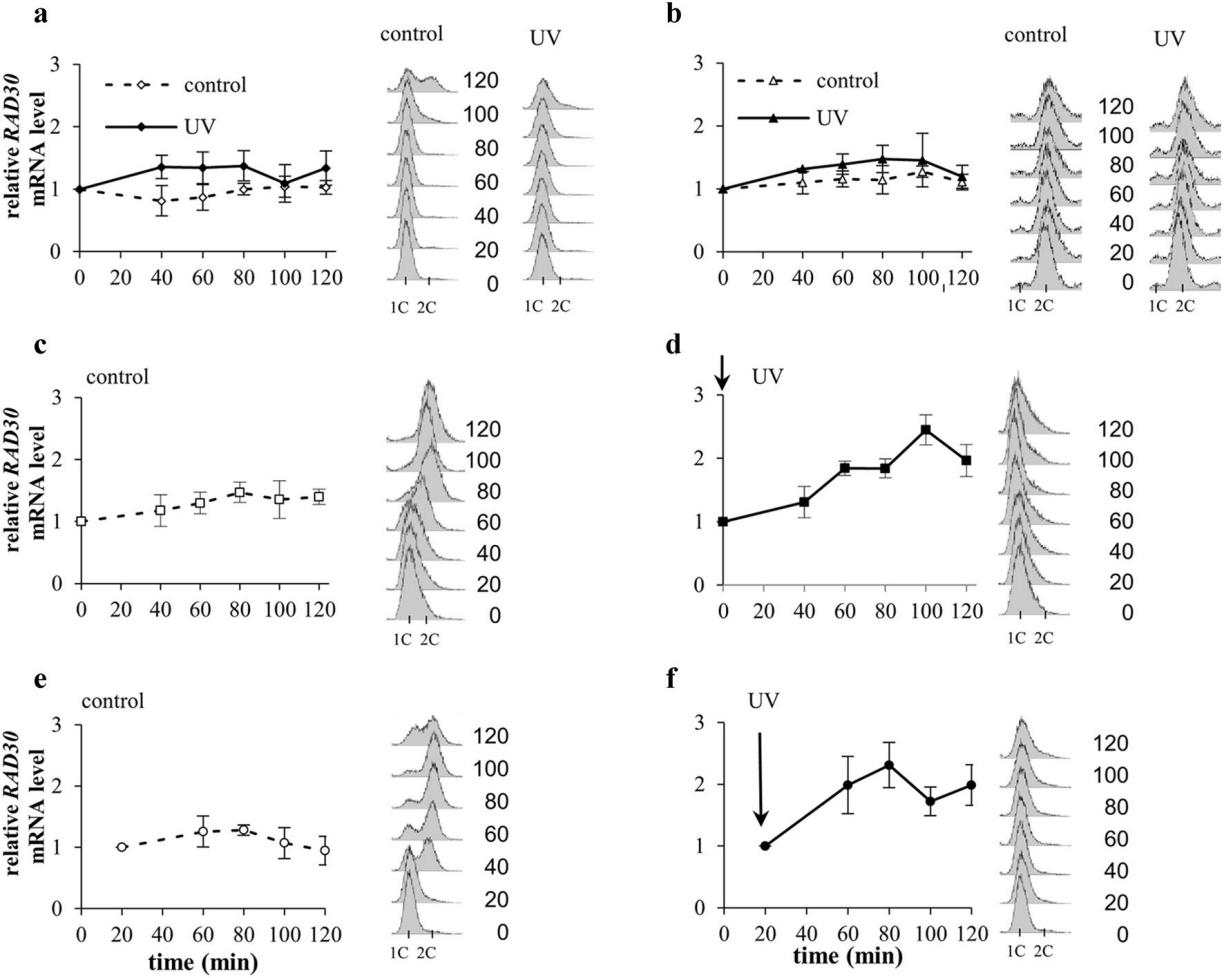
Relative levels of *RAD30* mRNA after UV irradiation (80 J/m^2^) at various stages of the cell cycle. **a** Cells were arrested in G1-phase with α factor (dashed line) and irradiated with UV (solid line). **b** Cells were arrested in G2/M-phase with nocodazole (dashed line) and irradiated with UV (solid line). **c**, **d** Cells were released from α factor arrest into S-phase progression (**c**, control) or irradiated with UV light immediately after release (time 0′) (**d**). **e**, **f** Cells were released from α factor arrest into S-phase progression (**e**, control) or irradiated with UV light 20 min after release (time 20′) (**f**). The presented data are the mean values from at least five independent experiments ± SDs. FACS data were used to monitor the DNA contents in control and irradiated yeast cells in G1-, G2/M-, or S-phase

The surprising finding that higher doses of UV radiation inhibited Pol eta and Rev1 accumulation in extended S-phase prompted us to investigate whether this effect was related to regulation of the levels of mRNAs encoding these polymerases. Previously published results of northern blot analyses have indicated that the level of *RAD30* mRNA encoding Pol eta is increased by three-to-fourfold in asynchronously growing yeast cultures irradiated with UV light at 80 J/m^2^ or higher doses (McDonald et al. 1997; Pabla et al. 2008; Roush et al. 1998). First, we investigated whether regulation of the *RAD30* mRNA level after UV radiation depends on the cell cycle stage. It has previously been shown that the abundance of *RAD30* mRNA under standard, stress-free conditions does not substantially change during the cell cycle (Plachta et al. 2015). When yeast cells were arrested in G1-phase with α factor and exposed to UV radiation (80 J/m^2^), a small increase (up to 50%) in the level of *RAD30* mRNA was observed 40–80 min after treatment (Fig. 4a). Similarly, small effects were detected after irradiation with lower UV doses (10 and 50 J/m^2^) (Supplementary material Fig. S4 a).

A weak tendency of the *RAD30* mRNA level to increase was also detected in cells synchronized in G2/M-phase with nocodazole and irradiated with 80 J/m^2^ (Fig. 4b) or 50 J/m^2^ but not in those irradiated with 10 J/m^2^ (Supplementary Fig. S4b).

In contrast, a significant increase in the mRNA level by over 2.5-fold was detected when cells were irradiated with 80 J/m^2^ at the beginning of S-phase (time 0′ after α factor removal) (compare Fig. 4c and d) and during ongoing S-phase 20 min after α factor removal (compare Fig. 4e and f). The lower doses of UV radiation (50 and 10 J/m^2^) caused similar or slightly lower increases in *RAD30* mRNA levels (Supplementary material Fig. S4c). These results indicated that the level of *RAD30* mRNA predominantly increased in yeast irradiated in S-phase of the cell cycle. The increasing doses of UV radiation gradually slowed S-phase progression which caused a gradual shift in *RAD30* mRNA accumulation to extended S-phase.

**Fig. 5:**
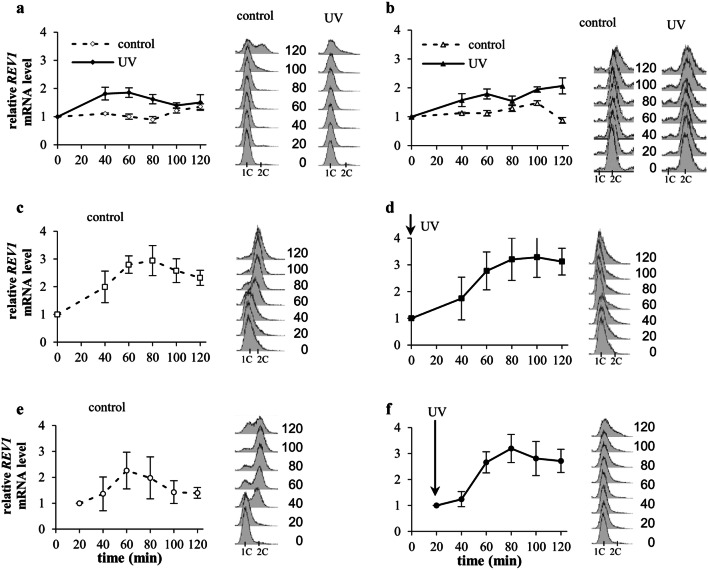
Relative levels of *REV1* mRNA after UV irradiation (80 J/m^2^) at various stages of the cell cycle. **a** Cells were arrested in G1-phase with α factor (dashed line) and irradiated with UV (solid line). **b** Cells were arrested in G2/M-phase with nocodazole (dashed line) and irradiated with UV (solid line). **c**, **d** Cells were released from α factor arrest into S-phase progression (**c**, control) or irradiated with UV light immediately after release (time 0′) (**d**). **e**, **f** Cells were released from α factor arrest into S-phase progression (**e**, control) or irradiated with UV light 20 min after release (time 20′) (**f**). The presented data are the mean values from at least five independent experiments ± SDs. FACS data were used to monitor the DNA contents in control and irradiated yeast cells in G1-, G2/M-, or S-phase

To determine whether mRNA accumulation in response to UV irradiation in cells arrested in S-phase is specific to *RAD30* mRNA and to elucidate whether the mRNAs of other Y-family polymerases accumulate in cells arrested in S-phase in response to UV irradiation, we determined the levels of *REV1* mRNA in yeast treated with UV radiation at various stages of the cell cycle. It has previously been shown that under stress-free conditions the level of mRNA encoding Rev1 is approximately threefold higher in G2/M-phase than in G1-phase (Waters and Walker 2006). Consistently, in control yeast cultures released to cell cycle progression after α factor removal, an almost threefold increase in the level of *REV1* mRNA was observed after 80 min of incubation (corresponding to G2-phase) in relation to the level at time 0′ (corresponding to the beginning of S-phase) (Fig. 5c). In relation to the start of S-phase, irradiation with various UV doses (80, 50 or 10 J/m^2^) in S-phase did not substantially affect the levels and timing of *REV1* mRNA accumulation (Fig. 5d, f, and Supplementary material Fig. S5c). However, extension of S-phase due to increasing UV doses caused a gradual shift in *REV1* mRNA accumulation from G2- to S-phase. Consistently, yeast cell irradiation with 80 J/m^2^ immediately after release from α factor arrest (time 0′) or 20 min later in S-phase (time 20′) arrested cell cycle progression at the beginning of S-phase or later in early S-phase (compare FACS data in Fig. 5c, d and in 5e, f), and after both treatments, we detected greater than threefold increases in the levels of *REV1* mRNA in S-phase-arrested cells (Fig. 5d, f). In contrast, UV irradiation (10, 50 or 80 J/m^2^) of cells arrested in G1-phase or G2/M-phase caused up to a twofold increases in the levels of *REV1* mRNA (Fig. 5a, b and Supplementary material Fig. S5a and b). We conclude that UV radiation increases the levels of mRNA encoding Rev1 in all phases of the cell cycle. However, after exposure to 80 J/m^2^, *REV1* mRNA, similar to Pol eta-encoding mRNA, accumulates predominantly in cells arrested in S-phase. This accumulation strongly suggests that the inhibitory effect of 80 J/m^2^ on Pol eta and Rev1 protein accumulation in S-phase-arrested cells is not regulated at the mRNA level.

### Accumulation of Pol eta and Rev1 in S-phase after HU treatment

Since irradiation with increasing doses of UV was correlated with a delay in S-phase progression, it could be assumed that the inhibition of polymerase accumulation could be correlated with inhibition of DNA synthesis.

**Fig. 6:**
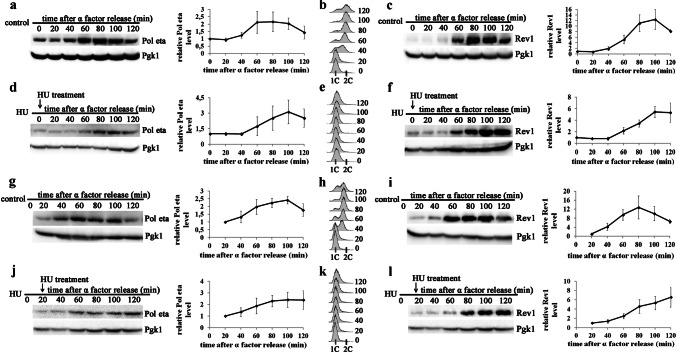
Levels of Pol eta and Rev1 in yeast arrested in S-phase by HU. Extracts from cells released from α factor arrest and harvested at the indicated time points were probed with an antibody against Pol eta (**a**, **d**, **g**, **j**) or Rev1-ProA (**c**, **f**, **i**, **l**) and antibody against Pgk1 as a loading control. Cells released into S-phase progression were treated with HU (150 mM) at the beginning of S-phase (time 0′) (**d**, **f**) or in 20 min later S-phase (time 20′) (**j**, **l**). **a**, **c**, **g**, **i** Untreated controls. **a**, **c**, **d**, **f**, **g**, **i**, **j**, **l** Western blots from representative experiments (left); the results are the mean quantified values from western blots from 3 to 4 independent experiments ± SDs (right). The band intensities were normalized to the intensities of the respective Pgk1 bands and to the intensities at time 0′ or 20. **b**, **e**, **h**, **k** FACS data were used to monitor S-phase progression

To determine whether the inhibition of Pol eta and Rev1 accumulation in cells arrested in S-phase is induced specifically by UV radiation at a high dose or reflects a more general response to agents that arrest DNA replication and activate the S-phase checkpoint, we analyzed the abundance of these two polymerases in yeast treated with HU. This compound is known to cause DNA replication stress by decreasing the pools of DNA replication precursors via inhibition of ribonucleotide reductase activity (Koç et al. 2004). The addition of HU to synchronized yeast cultures immediately after their release from α factor arrest blocked the progression of S-phase at its very beginning (Fig. 6e). In arrested cells, we observed the accumulation of both Pol eta and Rev1 (Fig. 6d, f). As in the untreated controls (Fig. 6a, c), polymerase accumulation in treated cells began after a 40-min lag, and resulted in a threefold and a sixfold increase in the levels of Pol eta and Rev1, respectively. When HU was added 20 min after the start of DNA replication, arresting ongoing S-phase, polymerase accumulation started without a delay, leading to comparable increases in Pol eta and Rev1 abundance in S-phase (Fig. 6j, l). These results suggested that HU treatment increased the potential for TLS in S-phase-arrested cells. On the other hand, our results indicate that DNA replication arrest is not sufficient to fully inhibit the accumulation of Y-family polymerases.

### Role of the S-phase checkpoint proteins, Mrc1 and Rad9, in regulating Y-family polymerase accumulation in response to UV radiation

**Fig. 7:**
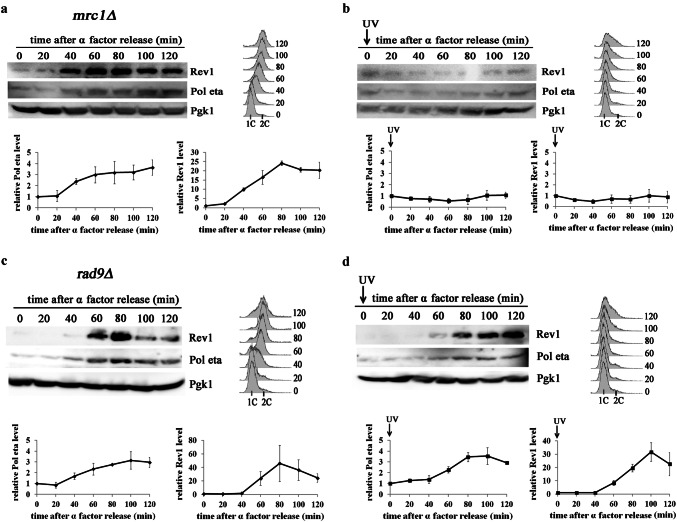
Effects of Mrc1 and Rad9 deficiency on the accumulation of Pol eta and Rev1 in cells arrested in S-phase in response to UV irradiation (80 J/m^2^). Extracts from cells released from α factor arrest and harvested at the indicated time points were probed with an antibody against Pol eta or Rev1-ProA and an antibody against Pgk1 as a loading control. Cells of *Δmrc1* or *Δrad9* mutant entering S-phase grew untreated (control; **a**, **c**) or were immediately irradiated with 80 J/m^2^ (**b, d**), Western blots from representative experiments (**a, b, c, d** top left), the results are the mean quantitative values from western blots from three independent experiments ± SDs for: Pol eta (**a, b, c, d** bottom left) or Rev1-ProA (**a, b, c, d** bottom right). Band intensities were normalized to the intensities of the respective Pgk1 bands and to intensities at time 0′. (**a, b, c, d** top right) FACS data were used to monitor S-phase progression

To identify the factors responsible for inhibiting Pol eta and Rev1 accumulation after exposure to higher doses of UV radiation, we investigated the role of the S-phase checkpoint in this process. It has recently been shown that S-phase checkpoint activation relies on two distinct mediators, Mrc1 and Rad9, which transmit the checkpoint signal from the sensor kinase, Mec1, to the main effector kinase, Rad53 (Pardo et al. 2017). In our study, we explored whether Mrc1- and/or Rad9 is responsible for regulating Pol eta and Rev1 accumulation in response to higher doses of UV radiation in S-phase. Treatment of cells devoid of either Mrc1 or Rad9 with UV radiation (80 J/m^2^) resulted in S-phase arrest, suggesting that both mediators can independently contribute to inhibition of S-phase progression in response to UV exposure (Fig. 7). Neither Mrc1 nor Rad9 deficiency had a significant effect on the accumulation of mRNAs encoding Pol eta and Rev1 in cells arrested in S-phase in response to this dose of UV radiation (Supplementary material Fig. S6). However, irradiation with 80 J/m^2^ largely abolished the accumulation of the polymerases in cells deficient in Mrc1 (Fig. 7b), similar to the situation in cells proficient in the S-phase checkpoint. In contrast, both Pol eta and Rev1 accumulation was detected in Rad9-deficient cells arrested in S-phase after UV irradiation with 80 J/m^2^ (Fig. 7d).

The accumulation of the polymerases after exposure to lower UV doses (10 and 50 J/m^2^) was not significantly higher in yeast cells deficient for the checkpoint proteins (Supplementary material Fig. S7) than in checkpoint-proficient controls. Therefore, we conclude that the presence of Rad9 (but not Mrc1) is responsible for the inhibition of Y-family polymerase accumulation in S-phase-arrested cells in response to high doses of UV radiation.

Additionally, our experiments indicated that Rad9-deficient cells exhibited over sixfold lower survival after irradiation with 80 J/m^2^ than cells proficient in S-phase checkpoint (6.9 ± 1.2% vs 48.2 ± 3.5% survival, respectively); in contrast, a difference in survival between Mrc1-deficient and control cells after this dose of UV was hardly detected (40.9 ± 7.9% vs 48.2 ± 3.5% survival, respectively). These results suggest that Rad9-dependent regulation of Y-family polymerase abundance in S-phase-arrested cells can contribute to better survival of yeast cells exposed to higher doses of UV radiation.

## Discussion

The presented results show a common pattern of regulation of Y-family TLS polymerase abundance in budding yeast treated with agents that damage DNA or disturb DNA replication at various stages of the cell cycle. Previous research on the cell cycle regulation of Rev1 (Waters and Walker 2006) and Pol eta (Plachta et al. 2015) levels under stress-free conditions has indicated similarities in the cell cycle regulation patterns of these two polymerases. However, a common regulatory strategy has seemed less obvious in cells treated with DNA-damaging agents, since Pol eta and Rev1 can play distinct roles in TLS and other processes linked to the maintenance of DNA stability (Prakash et al. 2005; Hirano and Sugimoto 2006; Acharya et al. 2019; Niu et al. 2019). Accordingly, in the promoter of the *RAD30* gene, which encodes Pol eta, two DNA damage responsive elements (*DREs*) have been recognized (McDonald et al. 1997), and increases in *RAD30* mRNA levels in response to UV radiation have been reported by several laboratories (McDonald et al. 1997; Pabla et al. 2008; Wiltrout and Walker 2011a, b). A response to DNA damage at the mRNA level has not been previously reported for the *REV1* gene. Instead, the levels of mRNA encoding Rev1 have been shown to fluctuate during the cell cycle and to be threefold higher in G2/M than in G1-phase in cells under stress-free conditions (Waters and Walker 2006). Such fluctuation has not been detected for *RAD30* mRNA (Plachta et al. 2015). The present results indicate that the increases in *RAD30* mRNA levels in response to UV radiation previously observed in asynchronous cultures reflect mainly mRNA accumulation in response to DNA damage induced in S-phase. This accumulation peaks 80–100 min after irradiation, and the peak corresponds to G2-phase in cells treated with low UV doses, which do not substantially slow S-phase progression. However, when S-phase progression is significantly prolonged or arrested, the same timing of mRNA accumulation places the accumulation peak in extended S-phase. Similarly, due to the UV radiation-induced S-phase progression delay, *REV1* mRNA accumulation shifts from G2-phase into extended S-phase after treatment with higher UV doses. As a result, both *RAD30* and *REV1* mRNAs accumulate in cells arrested in S-phase in response to UV irradiation with 80 J/m^2^. Intriguingly, this UV dose does not lead to substantial accumulation of the polymerases encoded by these mRNAs in S-phase-arrested cells. Our results indicate an inversely proportional relation between polymerase levels and UV dose, suggesting the existence of a regulatory mechanism that limits polymerase abundance in S-phase-arrested cells in response to DNA damage. Interestingly, a limitation of the Rev1 abundance in S-phase in response to DNA damage has also previously been shown to occur in fission yeast (Uchiyama et al. 2015). The authors suggested that this limitation can have a positive effect, since the artificial increases in Rev1 abundance in this phase result in elevated sensitivity to DNA damage. In budding yeast, the situation seems more complicated. Actually, two contradictory trends are clearly visible in budding yeast irradiated in S-phase. On one hand, increasing doses of UV radiation gradually slow the progression of S-phase. This delay, which is not accompanied by a change in the timing of polymerase accumulation in relation to S-phase entry, shifts Pol eta and Rev1 accumulation from G2-phase (in untreated cells) into extended S-phase. In parallel, increasing doses of UV radiation gradually limit the extent of the accumulation in delayed S-phase. The balance between these two trends determines the levels of Y-family polymerases after UV irradiation in S-phase. As a result, whereas irradiation with 50 J/m^2^ increases the levels of Pol eta and Rev1 in extended S-phase, increasing the TLS potential of this phase, the accumulation of the polymerases in extended S-phase is largely suppressed after irradiation with 80 J/m^2^.

In contrast to the inhibition of polymerase accumulation in cells arrested in S-phase after irradiation with 80 J/m^2^, nearly no limitation of Pol eta and Rev1 accumulation was observed in cells arrested in S-phase due to HU treatment. UV light and HU differ in the nature of their effects on DNA and cell responses. One of the important differences concerns the mechanism of activation of the S-phase checkpoint. Whereas HU treatment predominantly activates the DNA replication checkpoint (DRC), engaging the Mrc1 mediator, treatment with UV radiation and most other DNA-damaging agents primarily activates the DNA damage checkpoint (DDC), employing the Rad9-mediated pathway to phosphorylate Rad53 (Pardo et al. 2017). The functions of these two S-phase checkpoint subpathways have recently been established to be both temporarily and spatially separated (García‐Rodríguez et al. 2018; Bacal et al. 2018; Moriel-Carretero et al. 2019). Our results indicate that the limitation of Rev1 and Pol eta accumulation in S-phase-arrested cells after irradiation with 80 J/m^2^ depends on Rad9 but not Mrc1. How Rad9 is involved in regulating the accumulation of Y-family polymerases in cells arrested in S-phase remains an unanswered question. The results showing that UV exposure, which inhibited the accumulation of Pol eta and Rev1 in Rad9-proficient cells but did not similarly limit the accumulation of the mRNA encoding these polymerases strongly suggest that the mechanism that reduces polymerase abundance after UV irradiation operates at the protein level rather than at the mRNA level. Interestingly, polymerase abundance regulation in cells with undisturbed cell cycles has also been found to occur mainly at the protein level (Plachta et al. 2015; Wiltrout and Walker 2011a). One possible explanation is that the Rad9-dependent pathway somehow modifies this regulation. The Rad9 regulatory effect can be direct or indirect as the activity of this protein influences a number of cellular processes including DNA repair processes. First of all, Rad9 is a checkpoint kinase, and its function in S-phase is predominantly connected with DDC activation. It is quite possible that activation of S-phase checkpoint via the Rad9 pathway can directly or indirectly affect polymerase accumulation. Checkpoint activity has been reported to participate in the hyperphosphorylation of Rev1 (Sabbioneda et al. 2007). Rev1 was found to be modified in a Mec1-dependent manner in response to UV radiation and radiomimetic agents. Similar to the Rad9-dependent inhibition of Pol eta and Rev1 accumulation, Rev1 phosphorylation did not occur in response to HU treatment. However, in contrast to the regulation of Y-family polymerase abundance, the regulation of Rev1 hyperphosphorylation was not specific to S-phase. Additionally, Pol eta has not been shown to be phosphorylated in yeast in contrast to its mammalian homolog (Göhler et al. 2011). This suggests that Rev1 hyperphosphorylation is connected to a separate checkpoint-dependent mechanism that modulates the function of this enzyme and that the Rad9-dependent mechanism limiting Pol eta and Rev1 abundance in S-phase-arrested cells requires further investigation.

It is also worth noting that S-phase progression was arrested in response to UV exposure (80 J/m^2^) in the absence of either Rad9 or Mrc1. This result is consistent with previous findings, indicating that under certain conditions, DNA damage can activate both the DRC and DDC subpathways of the S-phase checkpoint (Bacal et al. 2018; García‐Rodríguez et al. 2018). Interestingly, although presence of Mrc1 or Rad9 alone was sufficient for S-phase progression arrest after exposure to 80 J/m^2^, the lethal effect of this treatment was much more pronounced in Rad9-deficient cells than in Mrc1-deficient cells, suggesting that Rad9-dependent regulation of the levels of Y-family polymerases may contribute to better cell survival in response to DNA damage.

Altogether, our results highlight a common strategy for the regulation of Y-family TLS polymerase abundance during the cell cycle in response to agents detrimental to DNA metabolism in budding yeast cells. This regulatory strategy is mainly applicable to cells treated in S-phase. Based on the differences in polymerase accumulation in cells arrested in S-phase in response to UV radiation and HU treatment and on the different involvement of Rad9 and Mrc1 checkpoint proteins in controlling this accumulation, we propose a speculative model of the role of the S-phase checkpoint in the regulation of cellular potential for TLS executed by Y-family polymerases. According to this model, in the context of S-phase extension in response to DNA replication stress, which activates the DRC, the TLS potential is increased by the accumulation of Y-family polymerases. Activation of the DDC by lower levels of DNA damage has a similar effect. However, further activation of the DDC subpathway via accumulation of DNA damage, e.g., after irradiation with UV at higher doses, suppresses TLS potential by limiting Y-family polymerase abundance in S-phase-arrested cells.

## Electronic supplementary material

Below is the link to the electronic supplementary material.

Supplementary file1 (DOCX 977 kb)
