# Supplementary material for: Regulation of the abundance of Y-family polymerases in the cell cycle of budding yeast in response to DNA damage
Source: Curr Genet. 2020 Feb 19;66(4):749–63. doi: 10.1007/s00294-020-01061-3 (PMC7363672; doi:10.1007/s00294-020-01061-3)
Supplement: Supplementary file 1 — Supplementary file1 (DOCX 977 kb) [file 294_2020_1061_MOESM1_ESM.docx]

**Supplementary Material**

**Current Genetics**

**Regulation of the abundance of Y-family polymerases in the cell cycle of budding yeast in response to DNA damage.**

Aleksandra Sobolewska, Agnieszka Halas, Michal Plachta, Justyna McIntyre and Ewa Sledziewska-Gojska

Institute of Biochemistry and Biophysics, Polish Academy of Sciences, 02-106 Warsaw, Poland

[esg@ibb.waw.pl](mailto:esg@ibb.waw.pl)

**
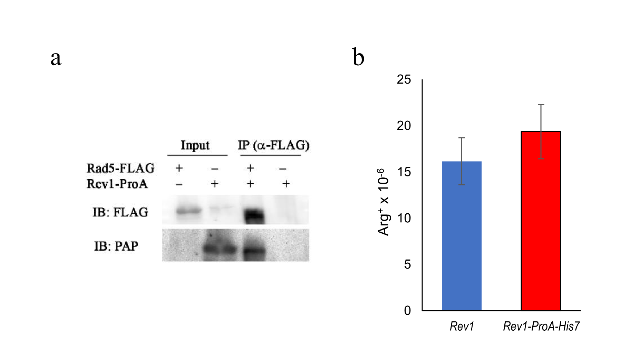
**

**Figure S1. ProA epitope does not change the Rev1 protein properties.** (**a**). Co-immunoprecipitation assay (IP) performed with anti-FLAG resins incubated with extracts from cells expressing Rad5 protein tagged with FLAG epitope and Rev1-ProA-His_7_ protein. (**b**) UV-induced reversion of the *arg4-17* mutation in strain producing native Rev1 and cells producing Rev1-ProA-His_7._ Reversion frequency was investigated in asynchronous cultures. The presented data are the mean values from three independent experiments ± SDs. Rev1-ProA-His_7_ protein interacts with Rad5-FLAG protein


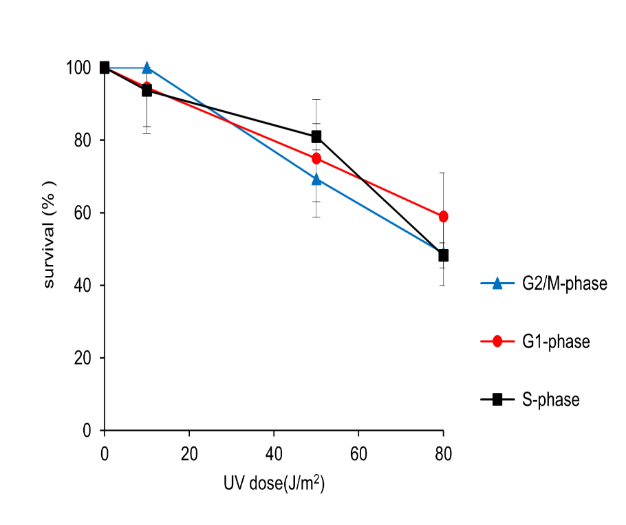


**Figure S2.** **Sensitivity of BY4741 to UV radiation at various phases of cell cycle.** Survival of cells exposed to indicated doses of UV radiation in G1-phase (arrested with α factor), in G2/M-phase (arrested with nocodazole), or immediately after release from α factor arrest for S-phase progression was calculated in relation to survival of untreated controls. The presented data are the mean values from three independent experiments ± SDs.





**Figure S3.** **The levels of Pol eta and Rev1 in yeast irradiated with UV light at entry into S-phase.** Extracts from cells released from α factor arrest and harvested at the indicated time points were probed with an antibody against Pol eta or Rev1-ProA, and an antibody against Pgk1 as a loading control. Cells entering S-phase grew untreated (control; **a**) or were immediately irradiated with 80 J/m^2^ (**b**) or 50 J/m^2^ **(c**). Western blots from representative experiments (**a, b, c** left); the quantification of Pol eta and Rev1 band intensities relative to respective Pgk1band intensities and those at time 0′ are shown at the bottom of each band (%). (**a, b, c** right) FACS data were used to monitor S-phase progression.

**
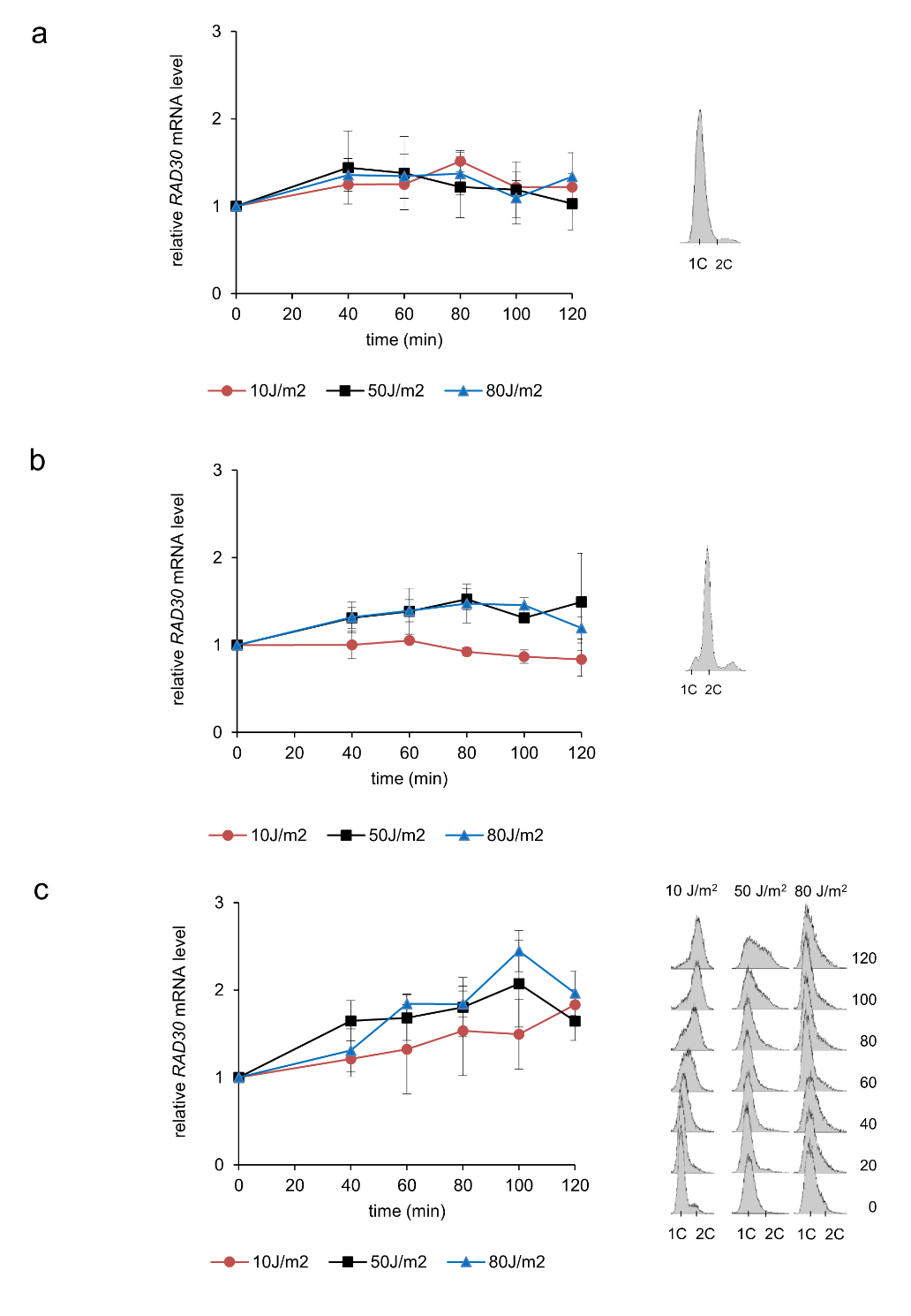
**

**Figure S4.** **Comparison of relative levels of *RAD30* mRNA in G1-, G2/M- and S-phase in response to various doses of UV.** Cells were arrested in G1 (with α factor) (**a**), G2/M (with nocodazole) (**b**), or released from α factor arrest into S-phase progression (**c**) and exposed to indicated doses of UV radiation. The presented data are the mean values from three independent experiments ± SDs. FACS data were used to monitor the DNA contents in control and irradiated yeast cells in G1-, G2/M- or S-phase.

**
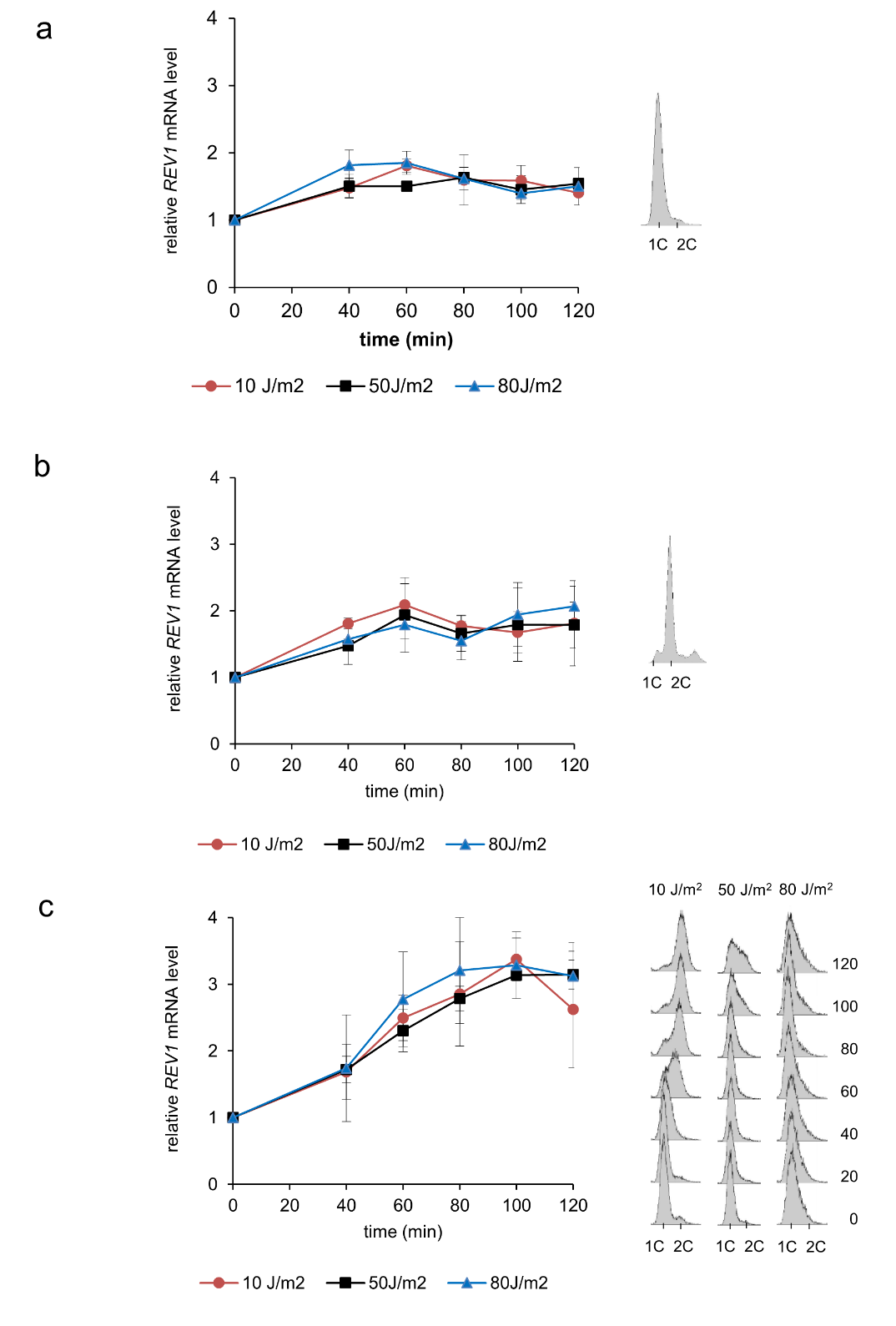
**

**Figure S5.** **Comparison of relative levels of *REV1* mRNA in G1-, G2/M- and S-phase in response to various doses of UV.** Cells were arrested in G1 (with α factor) (**a**), G2/M (with nocodazole) (**b**), or released from α factor arrest into S-phase progression (**c**) and exposed to indicated doses of UV radiation. The presented data are the mean values from three independent experiments ± SD. FACS data were used to monitor the DNA contents in control and irradiated yeast cells in G1-, G2/M- or S-phase.

**
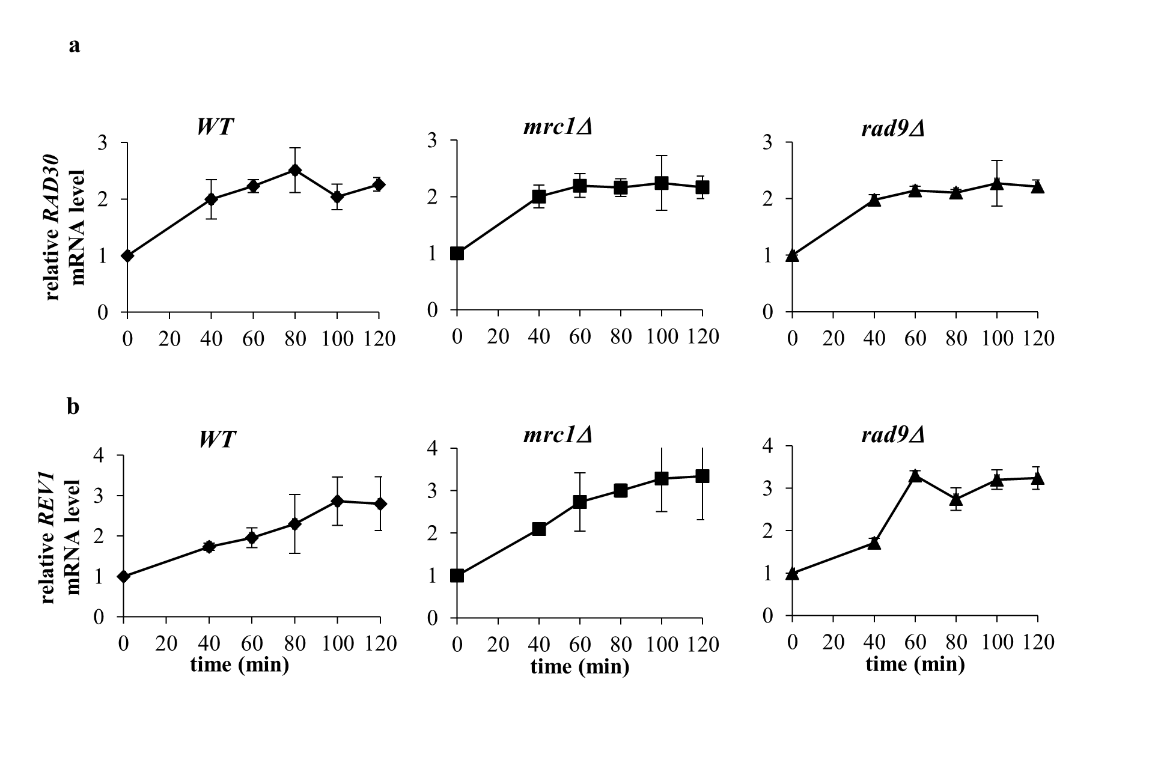
**

**Figure S6. The relative levels of Pol eta (a) and Rev1 (b) mRNA in Mrc1 or Rad9 deficient cells irradiated with UV (80J/m^2^) at the beginning of S-phase.** Cells were released from α factor arrest into S-phase progression and irradiated with UV light (80 J/m^2^) immediately after release (time 0′). The presented data are the mean values from three independent RT-qPCR experiments ± SDs. Corresponding FACS data, monitoring S-phase progression, are shown in the **FIG. 7**.





**Figure S7. The effects of Mrc1 and Rad9 deficiency on the accumulation of Pol eta and Rev1 after irradiation with 10 or 50 J/m^2^ in S-phase.** Extracts from cells released from α factor arrest, and harvested at the indicated time points, were probed with an antibody against Rev1-ProA or Pol eta and an antibody against Pgk1 as a loading control. (**a)**Western blots from representative experiments with extracts from *mrc1Δ* cells irradiated with 10 J/m^2^ (left) or 50 J/m^2^ (right) immediately after α factor removal. (**b**) FACS data monitoring S-phase progression in *mrc1Δ* cells irradiated with 10 J/m^2^ (left) or 50 J/m^2^ (right). (**c)**Western blots from representative experiments with extracts from *rad9Δ* cells irradiated with 10 J/m^2^ (left) or 50 J/m^2^ (right) immediately after α factor removal. (**d**) FACS data monitoring S-phase progression in *rad9Δ* cells irradiated with 10 J/m^2^ (left) or 50 J/m^2^ (right). The levels of Pol eta and Rev1 relative to Pgk1 and those at time 0′ are shown at the bottom of each panel
